# Ultrastructural and Descriptive Study on the Adult Body Surface of *Heortia vitessoides* (Lepidoptera: Crambidae)

**DOI:** 10.3390/insects14080687

**Published:** 2023-08-03

**Authors:** Lei Liu, Yan Zhang, Shan-Chun Yan, Bin Yang, Gui-Rong Wang

**Affiliations:** 1 Key Laboratory of Sustainable Forest Ecosystem Management—Ministry of Education, Northeast Forestry University, Harbin 150040, China; oinio@outlook.com (L.L.); givemefivezl@163.com (Y.Z.); 2 State Key Laboratory for Biology of Plant Diseases and Insect Pests, Institute of Plant Protection, Chinese Academy of Agricultural Sciences, Beijing 100193, China; wangguirong@caas.cn; 3 Guangdong Laboratory for Lingnan Modern Agriculture (Shenzhen Branch), Genome Analysis Laboratory of the Ministry of Agriculture, Agricultural Genomics Institute at Shenzhen, Chinese Academy of Agricultural Sciences, Shenzhen 518120, China

**Keywords:** ultrastructure, sexual dimorphism, sensilla, antenna, proboscis

## Abstract

**Simple Summary:**

The identification of the ultrastructure on the surface of insects serves as the foundation for understanding the molecular mechanisms underlying their perception of various environmental cues. In this study, we used scanning electron microscopy (SEM) to investigate the morphology, type, and distribution of sensilla on the antennae, mouthparts, and legs of the economically significant pest, *Heortia vitessoides*, which feeds on the valuable plant *Aquilaria sinensis*. Furthermore, we conducted a comparative analysis to assess the differences in sensillum characteristics, quantity, and distribution between male and female individuals.

**Abstract:**

*Heortia vitessoides* Moore, 1885 (Lepidoptera: Crambidae) is an economically important lepidopteran pest that caused severe damage to the plantation area of *Aquilaria sinensis* (Lour.) Gilg, 1825 (Thymelaeaceae), resulting in extensive defoliation of the trees during an epidemic. In this study, we used scanning electron microscopy (SEM) to analyze the external morphology and ultrastructure of sensilla on various body parts of *H. vitessoides*. Specifically, seven, four, four, and five types of sensilla were found, respectively, on the antennae, proboscis, labial palps, and legs. We described the types, distributions, and sexual dimorphism of these sensilla on antennae, and found that the number and size of sensilla differed significantly between males and females. This study provides crucial information for future investigations into the function of these sensilla in *H. vitessoides*.

## 1. Introduction

Insects rely on odorous chemical signals for essential behaviors, such as mate and habitat selection, communication, gathering, and hunting [1,2,3]. To accurately detect and interpret these signals, insects have evolved a sophisticated sensory system wherein odor molecules travel through sensilla pores and activate sensory neurons. These neurons subsequently transform these chemical signals into electrical signals, which ultimately generate action potentials [4]. Sensilla serve as the primary point of contact for insects to perceive and interpret their surroundings, and are thus a crucial sensory component in perceiving chemical pheromones [5]. Examining the morphology and structure of sensilla in adult moths is fundamental to investigations on sensory recognition mechanisms and insect behavior.

Lepidoptera, the second largest order of insects [6], possess diverse sensilla on their antennae, proboscis, labial palps, and legs. The antennae serve as the primary sensory organs and are essential for mechanoreception, gustation, olfaction, temperature and humidity perception, and CO_2_ recognition in Lepidoptera [7]. The mouthparts, including the proboscis and labial palps, have a multitude of sensilla that are vital for locating food sources, host identification, and selection of oviposition sites [8,9,10,11,12]. Moreover, the sensilla in the labial palp pit organ (LPPO) have been reported to respond to a variety of plant-derived odors and are sensitive to CO_2_ in several species of moths [13,14,15]. Although the tarsal segments of the thoracic legs have fewer and different types of sensilla compared to the antennae and mouthparts, electrophysiological studies suggest that they are involved in gustatory functions [16]. Additionally, it is proposed that the legs may also function in gustation, olfaction, and in sensing temperature, humidity, and gravity; however, these hypotheses remain to be confirmed.

*Heortia vitessoides* Moore, 1885 (Lepidoptera: Crambidae) is an oligophagous insect that predominantly infests *Aquilaria sinensis* (Lour.) Gilg, 1825 (Thymelaeaceae) [17]. *A. sinensis* produces agarwood, a natural resin that is produced in response to injury, which has high economic value in China due to its use as a precious medicinal material and natural spice [18,19]. *H. vitessoides* is distributed throughout tropical and subtropical regions, including Southeast Asia and Oceania [20,21]. Based on the analysis of climatic factors and model construction, Xu et al. predicted that a suitable growing region for *H. vitessoides* in China would expand northward [22]. As the artificial planting area of *A. sinensis* continues to expand, larvae of *H. vitessoides* have become increasingly destructive and caused significant economic losses. They can aggregate and quickly consume leaves within days (Figure S1H), and when food is scarce, they can even eat the epidermis of branches [23]. The depletion of leaves limits the use of whole-body fragrance technology, which is reliant on transpiration [24], causing a significant hindrance to the tea industry that uses *A. sinensis* leaves as raw materials in some planting areas of Guangdong. Given the high medicinal value of *A. sinensis*, efficient and environmentally sound control measures for this devastating insect pest are urgently needed. Much research has already been conducted on the biological characteristics and chemical control strategies of *H. vitessoides* [25], and a variety of chemosensory genes have been identified via transcriptome sequencing of antennae and mouthparts [26]. Qiao et al. conducted an ultrastructural of the antennae of *H. vitessoides*, in which they identified and described different types of sensilla [27]. The availability of a complete genome sequence for *H. vitessoides* has facilitated comparative genomic studies that have greatly expanded our understanding of species’ phylogenetic relationships and evolutionary history [28]. Although volatile components produced by *A. sinensis* that elicit electrophysiological activity in *H. vitessoides* sensilla have been identified using electroantennography (EAG) [20], the precise molecular mechanism responsible for chemical pheromone recognition remains unclear.

In this study, we meticulously examined and documented the morphology, types, and distribution of sensilla on the antennae, mouthparts, and legs of adult female and male *H. vitessoides* using scanning electron microscopy (SEM). Furthermore, the variations in sensilla characteristics, abundance, and distribution between the male and female specimens were comparatively analyzed. The findings of this study may help clarify the molecular mechanisms underlying chemosensation in *H. vitessoides* and establish a basis for further research into insect–insect or plant–insect pheromone interactions. These results may also provide theoretical guidance for the development of environmentally sound insect pest technologies.

## 2. Results

### 2.1. Morphological Structure of the Antennae

**Figure 1:**
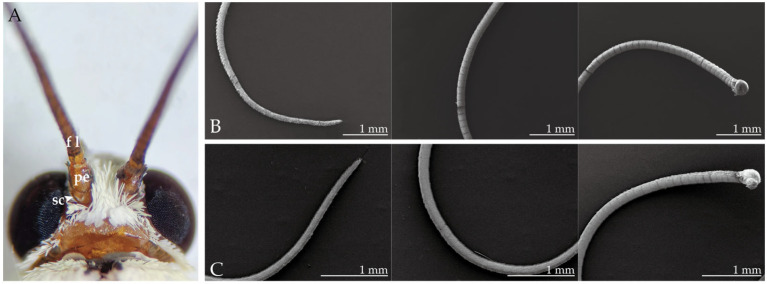
General morphology of the antennae of *H. vitessoides* (**A**) An aerial perspective of the antennae, comprising three distinct segments: the scape (sc), pedicel (pe), and flagellum (fl); (**B**) overall antennal structure of the male; and (**C**) overall antennal structure of the female. Figure (**B**,**C**) illustrate the distal, middle, and basal parts of the antennae from left to right.

The filamentous antenna of female and male *H. vitessoides* consists of a basal scape, a pedicel, and an elongated flagellum with more than 65 flagellomeres (Figure 1). Scales and sparse sensilla are distributed on the first two parts and the dorsal surface of the flagellum, while the ventral surface of the flagellum lacks scales and is instead equipped with ample chemosensory sensilla. The flagellum cuticle is a structural network composed of polygonal and circular patterns; compared with the distal end of the flagellum, chemosensory sensilla are denser in the middle and, especially, the basal portions of the flagellum. However, there is a greater variety of sensilla types at the distal end, and the flagella of female *H. vitessoides* are significantly longer than in males (female: 8.9 ± 0.20 mm length; male: 7.3 ± 0.26 mm length). While the number of sensilla styloconica in the last segment of the antennal flagellum in males was greater than in females, there were no other significant differences observed between the sexes.

### 2.2. Antennal Sensilla in H. vitessoides

A total of seven sensilla types and four subtypes were observed in both male and female *H. vitessoides*. These included sensilla trichodea I and II, sensilla coeloconica I and II, sensilla chaetica, sensilla styloconica, sensilla basiconica I and II, capitate sensilla basiconica, sensilla squamiformia, and Böhm bristles. Table 1 lists the observed sexually dimorphic features, and each of the identified sensilla types is described in detail in the subsequent paragraphs.

**Figure 2:**
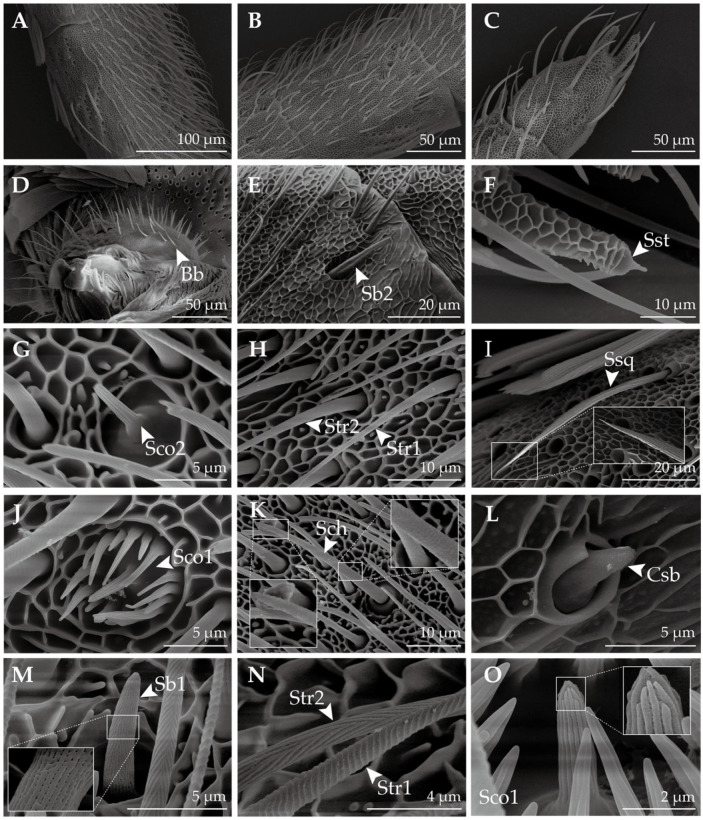
Ultrastructure of the antennae of *H. vitessoides.* (**A**) The basal parts of the flagellum; (**B**) the middle parts of the flagellum; (**C**) the distal parts of the flagellum; (**D**) Böhm bristles (Bb); (**E**) sensillum basiconicum II (Sb2); (**F**) sensillum styloconicum (Sst); (**G**) sensillum coeloconicum II (Sco2); (**H**) sensillum trichodeum I (Str1); sensillum trichodeum II (Str2); (**I**) sensillum squamiformium (Ssq); (**J**) sensillum coeloconicum I (Sco1); (**K**) sensillum chaeticum (Sch); (**L**) capitate sensillum basiconicum (Csb); (**M**) sensillum basiconicum I (Sb1); (**N**) wall pattern of Str1 and Str2; and (**O**) dense sensilla pore on Sco1.

Sensilla trichodea (Str, Figure 2H,N), which represent the most prevalent sensory structures on the antennae of both male and female *H. vitessoides*, are primarily found on the ventral aspect of the flagellum. These structures are predominantly distributed on the basal segment of the flagellum, followed by the middle and distal parts. Multiporous Str are hair-like structures, decreasing in diameter from the base to the tip, lacking a cupping depression at the base, and featuring a threaded surface. Sensilla trichodea II (Str2) are comparatively shorter than sensilla trichodea I (Str1); Str1 form an annular pattern, while Str2 form a vertically oriented twill pattern on the wall. Additionally, Str2 are sparsely distributed amongst Str1.

Sensilla basiconica I (Sb1, Figure 2M) are sparsely dispersed on the ventral surface of the flagellum, exhibiting a morphology that bears a resemblance to Str. These sensilla are vertical or slightly curved on the surface and devoid of a cup-shaped fossa at the base. Nonetheless, Sb1 are thicker and shorter than Str. Additionally, they possess an obtusely rounded apex and a multiporous surface. Sensilla basiconica II (Sb2, Figure 2E) and capitate sensilla basiconica (Csb, Figure 2L) are distributed on both sides of the antennae near the internodes of the flagellum, with only a few of these sensilla being found on the distal and middle parts of the flagellomere. Aporous Sb2 feature a slightly pointed tip that exhibits near-parallel orientation to the cuticle, and the sensilla possesses a concave shape that curves inward from the sides towards the center. Aporous Csb are characterized by a hemispherical-like appearance, having a smooth surface and obtusely round tip, and are inserted in a circular cavity in depressions surrounded by a smooth thick cuticle.

Multiporous sensilla coeloconica I (Sco1, Figure 2J,O) are characterized by a central peg encircled by 8–14 arched spines that curve inwards towards the center, forming a chrysanthemum-like shape. Conversely, sensilla coeloconica II (Sco2, Figure 2G) are solely comprised of a central peg with a multiporous stripe wall, and are devoid of any spines. Sensilla chaetica (Sch, Figure 2K) are sparsely distributed on each flagellomere, with the highest concentration located on the distal parts. These aporous sensilla are rigid and sharp, featuring an obtusely rounded apex and circular patterns on the wall. Sch are thicker and longer than Str, and are situated in a protruding, cup-shaped fossa, which distinguishes them from Str.

**Figure 3:**
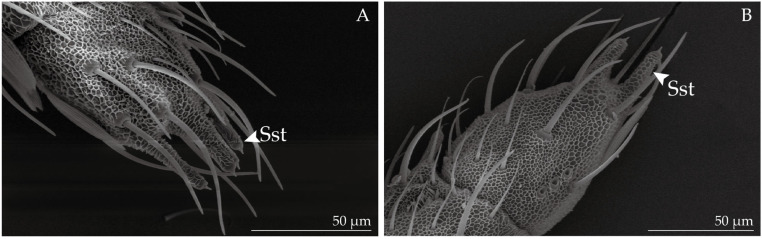
The numbers of sensilla styloconica (Sst) in the last flagellomere at the distal end of the male flagellum (**A**) are higher than that of the female (**B**).

Sensilla styloconica (Sst, Figure 2F) are the thickest of all the sensilla (Table 1). These aporous sensilla are distributed on the sides of the distal parts of each flagellomere, with several—two in females and four in males—being located at the top of the last flagellomere (Figure 3). Sst is characterized by a thumb-shaped structure with a small cone on top, lacks a cup-shaped fossa at the base, and has a reticulate pattern on the wall which is the continuation of the cuticle. Sensilla squamiformia (Ssq, Figure 2I) are present on the back of the flagellum, and are often covered with scales. These sensilla exhibit a scale-like morphology with longitudinal ridges, featuring a slender, fusiform shape that widens in the middle and tapers towards both ends, curving downward from the midpoint. Their tops are near the surface of the antennae, and are situated in protruding circular cup-shaped fossa. Böhm bristles (Bb, Figure 2D) cluster along the basal parts of the scape and pedicel. Unlike other sensilla, Bb are not found on the elongated flagellum of the antennae. Bb structures are oriented perpendicular to the surface of the antennae, resembling short thorns that are shorter and more pointed than Sch. Furthermore, Bb lack a cup-shaped fossa and possess a smooth surface absent of pores.

### 2.3. Morphological Structure of the Mouthparts

Similar to other lepidopteran species, the proboscis and labial palps are the primary organs required for gustatory perception in *H. vitessoides*. These mouthparts are located between the two compound eyes, and while in a resting state, the proboscis is coiled in a spiral shape and connected to the labial palps on both sides. During feeding or probing, the proboscis extends fully, assuming a long, tubular shape, while the labial palps protrude slightly.

**Figure 4:**
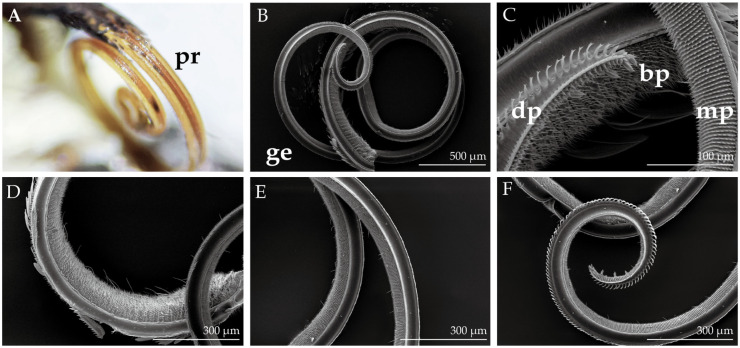
General morphology of the proboscis of *H. vitessoides.* (**A**) Coiled proboscis (pr) in resting state; (**B**) overall structure of one side elongated galeae (ge); (**C**) the sharp distal parts (dp), the rough basal parts (bp), and the neat middle parts (mp); (**D**) the basal parts of the proboscis; (**E**) the middle parts of the proboscis; and (**F**) inner side of the distal parts of the proboscis.

**Figure 5:**
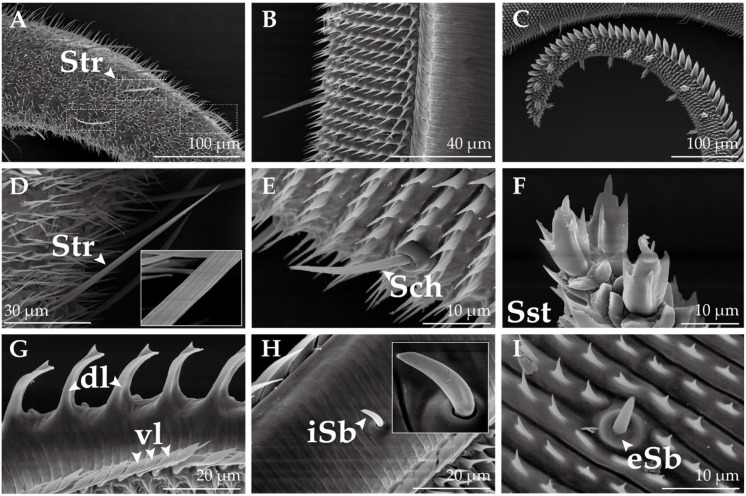
Ultrastructure of the proboscis of *H. vitessoides*. (**A**) Sensilla trichodea (Str) at the basal parts of elongated galeae; (**B**) the middle parts of elongated galeae; (**C**) the distal parts of elongated galeae; (**D**) texture on the surface of sensillum trichodeum (Str); (**E**) sensillum chaeticum (Sch); (**F**) sensilla styloconica (Sst) at the end of elongated galeae; (**G**) dorsal legulae (dl) and ventral legulae (vl); (**H**) internal sensillum basiconicum (iSb); and (**I**) external sensillum basiconicum (eSb).

**Figure 6:**
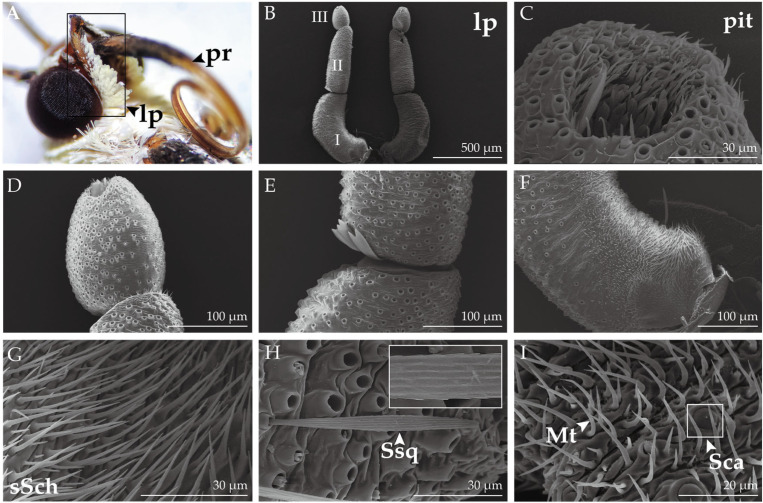
Ultrastructure of the labial palps of *H. vitessoides*. (**A**) Labial palps (lp) on both sides of the proboscis (pr); (**B**) overall structure of labia palps (lp); (**C**) Labial palp pit organ (LPPO); (**D**) the distal parts of labial palps; (**E**) the middle parts of labial palps; (**F**) the basal parts of labial palps; (**G**) short sensilla chaetica (sSch); (**H**) sensillum squamiformium (Ssq); and (**I**) the arrow points to the microtrichia (Mt) and the white box frames the sensillum campaniformium (Sca).

The proboscis (Figure 4) comprises two elongated galeae that gradually taper from the base to the distal end, which are connected by petal-like dorsal legulae and curled ventral legulae (Figure 5G) to form a capillary structure for siphoning liquids. The probosci’s surface is adorned with densely populated cuticular processes, exhibiting a gradual reduction in length as they extend from the base. The surface of the labial palps is covered with dense scales that are c-shaped and upwardly curved. Upon removal of the scales, three segments of the labial palps (Figure 6B) can be seen, with the first segment being the longest and the third segment having an oval depression at the top, known as the labial palp pit organ (LPPO, Figure 6C).

### 2.4. Proboscis and Labial Palps Sensilla in H. vitessoides

A total of four types and one subtype of sensilla were identified on the proboscis of *H. vitessoides*. Sensilla styloconica (Sst, Figure 5F) are the most common type of sensilla found on the proboscis of lepidopteran species and are exclusively located at the distal end of the proboscis. Sst possess a columnar projection at the base, with five to six ridged ribs that extend upward to form a spine-like structure. The base of the sensilla are depressed in the cuticle and project a short, conical sensory cone that is short in with a smooth surface, and a lotus-like pedestal. 

Sensilla basiconica (Sb) on the proboscis are classified into two subtypes. External sensilla basiconica (eSb, Figure 5I) protrude perpendicularly from the outer cuticle of the proboscis and have a short and conical shape, featuring a smooth and bluntly rounded surface at the top and a cup-shaped fossa at the bottom. These sensilla are distributed only on the distal and middle parts of the proboscis. Internal sensilla basiconica (iSb, Figure 5H) are arranged in regular rows and distributed from the base to the distal parts of the inner surface of the proboscis, and are more elongated than the eSb. The basal fossa is slightly convex relative to the inner cuticle, with the surface being smooth but more apically acute. Sensilla trichodea (Str, Figure 5D) are densely distributed exclusively on the basal parts. These aporous sensilla gradually narrow from the base to the top, have vertical lines on the surface, and lack a fossa at the base. Sensilla chaetica (Sch, Figure 5E) are distributed on the middle and basal parts of the proboscis and have a similar appearance to the Str. However, the aporous Sch are shorter and thicker, erect at an angle to the surface of the proboscis, and have a cup-shaped fossa at the base.

A total of four sensilla types were identified on the labial palps of *H. vitessoides.* Short sensilla chaetica (sSch, Figure 6G) are clustered in large numbers at the base of the first segment and at the junction of the second and third segments. These sensilla are short-spined, oriented perpendicular to the surface of the labial palps, and have a smooth, non-porous surface. sSch are encircled by a multitude of hair-like microtrichia (Mt, Figure 6I), feature a stout and tapered base, and curve at a wide angle. Sensilla campaniformia (Sca, Figure 6I) were found exclusively on the inner side of the first segment. Each sensillum consists of a ring of slightly raised cuticles surrounding a dome-shaped structure with a raised dot at the center of the dome surface. Sensilla squamiformia (Ssq, Figure 6H) were found exclusively on the second segment and are morphologically similar to the corresponding type of sensilla found on the antennae.

### 2.5. Morphological Structure of the Leg

**Figure 7:**
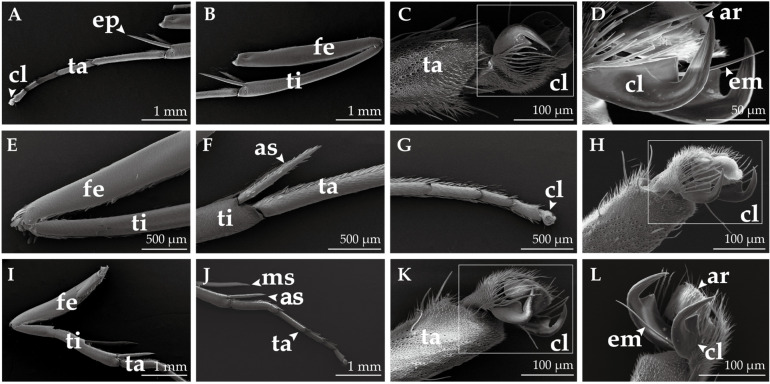
General morphology of the leg of *H. vitessoides.* (**A**–**D**) Foreleg; (**E**–**H**) midleg; (**I**–**L**) hindleg. claw (cl); tarsus (ta); tibia (ti); femur (fe); epiphysis (ep); apical spurs (as); medial spurs (ms); empodium (em); arolium (ar). The boxed areas in Figure (**C**,**H**,**K**) are claws (cl).

**Figure 8:**
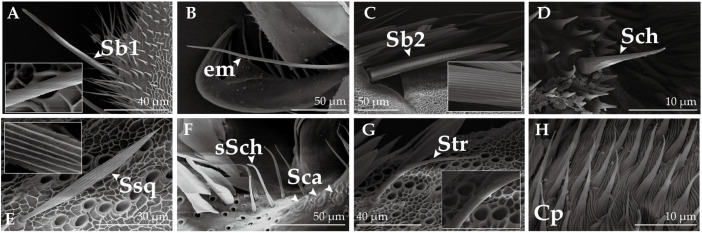
Ultrastructure of the Legs of *H. vitessoides*. (**A**) Sensillum basiconicum I (Sb1); (**B**) empodium (em); (**C**) sensillum basiconicum II (Sb2); (**D**) sensillum chaeticum (Sch); (**E**) sensillum squamiformium (Ssq); (**F**) sensilla campaniformia (Sca) and short sensilla chaetica (sSch); (**G**) sensillum trichodeum (Str); (**H**) cuticular processes (Cp).

The foreleg, midleg, and hindleg of *H. vitessoides* are situated on the lateral ventral surfaces of the prothorax, mesothorax, and metathorax, respectively. Each leg comprises five distinct parts, including the coxa, trochanter, femur (fe), tibia (ti), and tarsus (ta). The femur is the most robust, while the tibia of the foreleg bears an epiphysis (ep, Figure 7A) on its distal ventral surface. The tibia of both the midleg and hindleg has a pair of apical spurs (as, Figure 7F,J); the hindleg tibia bears an additional pair of medial spurs (ms, Figure 7J) at its midpoint, rendering it longer than the other legs. The tarsus is itself composed of five segments, with the basitarsus being the longest, followed by a progressively shortening series of tarsomeres, and the pretarsus is slightly longer than the fourth tarsite. The pretarsus comprises one pair of lateral claws (cl), which are curved forward and possess longitudinal ribs on their surface, as well as a slender empodium and arolium (ar) located between the cl. Notably, the lateral cl and ar are surrounded by a large cuticle containing numerous spine-like processes (Cp, Figure 8H), which gradually increase in length from the base upwards.

### 2.6. Leg Sensilla in Adult H. vitessoides

A total of five types and two subtypes of sensilla were identified on the legs of *H. vitessoides*, with no differences in the type of sensilla being observed on the forelegs, midlegs, and hindlegs. Among these, sensilla basiconica I (Sb1, Figure 8A) were found to be the most abundantly distributed on the tarsus, exhibiting a gradual taper from the base, and terminating with a rounded apical end. Sb1 are situated in a sunken position at the base and lack a fossa, while the surface is marked by linear ridges resembling grains of corn. Sensilla basiconica II (Sb2, Figure 8C) are situated at the ventral end of each tarsite and are sturdier than the other types, possessing a semi-enclosed fossa and a vertical stripe on the surface. Sensilla chaetica (Sch, Figure 8D) are situated at the junction of the tarsus and claw, taking the form of a cone with a textured elevated surface. Sensilla trichodea (Str, Figure 8G) are sparsely distributed and located on the outer cuticle, exhibiting a slightly upwardly directed apex. The morphology of Str are similar to the corresponding type of sensilla observed on the antennae, but its surface pattern has a distinct vertical pattern. Sensilla squamiformia (Ssq, Figure 8E) are distributed on the femur, tibia, and tarsus of the midlegs and hindlegs, with the highest density being found on the tarsus. Conversely, on the foreleg, this type of sensilla is present solely on the tarsus. The morphology of these sensilla types are strikingly similar across all body parts. Short sensilla chaetica and sensilla campaniformia (sSch and Sca, Figure 8F) are located at the junction of the femur and tibia; these sensilla are morphologically similar to those observed in the labial palps, but sSch are sparser on the legs.

## 3. Discussion

In this study, we used scanning electron microscopy (SEM) to observe and identify sensilla on the antennae, mouthparts, and legs of adult *H. vitessoides.* Overall, we were able to identify a total of seven types and four subtypes of sensilla on the antennae of *H. vitessoides*, which included sensilla trichodea I and II, sensilla coeloconica I and II, sensilla chaetica, sensilla styloconica, sensilla basiconica I and II, capitate sensilla basiconica, sensilla squamiformia, and Böhm bristles. The structure and types of these sensilla were similar to that reported in other lepidopteran species: *Tuta absoluta* Meyrick, 1917 [29], *Diaphania angustalis* Snellen, 1895 [30], *Coleophora obducta* Meyrick, 1931 [31], *Cnaphalocrocis medinalis* Guenée, 1854 [32] *Helicoverpa assulta* Guenée, 1852 and *Helicoverpa armigera* Hardwick, 1965 [33], and all other lepidopterans [34]. Nonetheless, slight variations existed with respect to their quantity, distribution, and external morphology. Sensilla trichodea, which are the most abundant and extensively dispersed on the flagellum of *H. vitessoides*, serve as the primary sensilla for detecting interspecific sex pheromones [33,34,35] and plant volatiles [36,37]. Generally, sensilla trichodea are more abundant in male moths, a common trait observed in lepidopteran species [30,34,38]. Nevertheless, in *H. vitessoides*, there is a noticeable disparity in length between the sensilla trichodea II of females and males. Additionally, sensilla trichodea II are comparatively shorter and less abundant than sensilla trichodea I. These findings raise speculation that sensilla trichodea I might be implicated in recognition of sex pheromones. Regrettably, we did not observe the presence of sensilla auricillica in our study. However, Qiao et al. reported the discovery of this type of sensilla in their observations [27], and reported that their morphology bears resemblance to those in Pyralidae moths [32,39], which are known to assist female moths in detecting host plant volatiles and male moths in detecting sex pheromones [40,41,42]. Sensilla basiconica, which are present in smaller numbers compared to sensilla trichodea, are also thought to be involved in the perception of both plant volatiles and sex pheromones [32,43,44]. Aporous sensilla chaetica, the longest sensilla on the *H. vitessoides* antennae, are mainly distributed on the distal parts’ last few segments and are believed to be responsible for mechanical sensation [34,45,46]. In *Periplaneta americana* Linnaeus, 1758, sensilla chaetica had both tactile and gustatory functions [47], while electrophysiological studies have demonstrated that they can be activated by sugars and alkaloids [48]. Therefore, it is suspected that the sensory neurons in sensilla chaetica of *H. vitessoides* express sugar-recognizing gustatory receptors, allowing them to sense liquids and initiate proboscis protrusion for feeding. In Hymenoptera, sensory neurons in sensilla coeloconica have been found to possess a large number of evolutionarily conserved ionotropic receptors, indicating their possible involvement in the recognition of acids, amines, and aldehydes and their similar functions across species [49]. Aside from their olfactory functions, these sensilla are sensitive to temperature, humidity, heat, and CO_2,_ and also serve as an anti-desiccation mechanism [50,51]. Aporous antennal sensilla styloconica are also capable of sensing temperature and humidity [34,39,52]. Sensilla squamiformia are probably tactile mechanoreceptors [34]. Böhm bristles are a type of mechanical stimuli sensing sensilla that are prevalent in insects. They have the ability to sense gravity and regulate the position of moth antennae through a reflection mechanism, thereby influencing the direction and angle of flight [53].

The mouthparts of *H. vitessoides* are similar to those of other Lepidoptera, *H. armigera* [8], *Spodoptera frugiperda* Smith and Abbot, 1797 [13], *Plutella xylostella* Linnaeus, 1758 [9], and all other lepidopterans [34]. The proboscis is the sole organ for adult moths to acquire nutrients, and it is used to collect dew and nectar [54]. The two labial palps are located on both sides of the proboscis and can monitor fluctuations in CO_2_ content in the surrounding environment, allowing insects to detect CO_2_ emitted by plants and locate nectar [12,55]. The sensilla on the proboscis and labial palps of *H. vitessoides* were identified for the first time, and seven types of sensilla were found on the mouthparts: sensilla trichodea, sensilla basiconica, sensilla chaetica, sensilla styloconica, sensilla squamiformia, sensilla campaniformia, and microtrichia. Uniporous sensilla styloconica are a common type of sensilla found throughout the lepidopteran proboscis [56]. These sensilla are capable of sensing a wide range of chemicals, including sugars, amino acids, and nicotine [57]. In addition to their chemosensory capabilities, they serve a simple mechanical function: dense clusters of sensilla styloconica located at the end of the proboscis are used by moths to collect pollen [12]. Sensilla basiconica have been classified into two subtypes based on their location within the internal and external cuticle of the proboscis. It is believed that these types of sensilla are involved in the detection of liquid flow velocity during feeding, and they have been shown to exhibit an electrophysiological response to sucrose solutions [58]. Sensilla chaetica have a similar shape to sensilla trichodea. In *H. vitessoides*, sensilla trichodea is exclusively found in the basal parts of the proboscis, which is different from their distribution in *P. xylostella* [9]. Aporous sensilla trichodea are typically involved in sensory functions and can provide information on depth and other metrics during nectar siphoning [59]. The labial palps of female *H. vitessoides* are longer than that of males, and this sexual dimorphism also exists in *S. frugiperda* [13], *Mythimna separata* Walker, 1865 [60], *Cactoblastis cactorum* Berg, 1885 [61] and *P. xylostella* [9]. The elongated labial palps of female *H. vitessoides* are believed to aid in host plant exploration, which may be linked to female reproductive selection. The findings obtained through single sensillum recording (SSR) experiments demonstrate that the uniporous sensilla chaetica located on the labial palps exhibit responsiveness to salt and sugar stimuli, indicating their potential gustatory sensory functions in addition to their role in mechanoreception [62], while sensilla campaniformia are believed to be involved in sensing cuticle deformation [63]. The labial palp organ (LPPO), containing numerous flattened sensilla that are responsible for the detection and identification of CO_2,_ serves as the chemical sensory center of the labial palps [13,64]. Regrettably, the internal structure of the LPPO was not fully observable in this study, necessitating further investigations using transmission electron microscopy.

In contrast to the antennae and mouthparts of moths, there has been a paucity of ultrastructural observations and functional studies on the leg sensilla [34]. Five types and two subtypes of sensilla have been identified on the legs of *H. vitessoides*: sensilla basiconica I and II, sensilla chaetica, short sensilla chaetica, sensilla squamiformia, sensilla trichodea and sensilla campaniformia. These sensilla are present on all legs and are primarily localized on the tarsus, with the highest density occurring at the distal end of the tarsus. They are responsible for functions such as touch, olfaction, mechanoreception, and graviception. The tarsus of lepidopteran moths is also believed to perform gustatory functions. Electrophysiological research has revealed that the sensilla on the tarsus are capable of detecting sugars, salts, amino acids, and bitter compounds [16,65], which may be necessitated in adult feeding.

## 4. Materials and Methods

### 4.1. Insect

Egg masses of *H. vitessoides* were collected in September 2021 from DongShan Tsuen (22°44′ N, 109°55′ E) in the Guangxi Province and Feng Guang Aquilaria Professional cooperative (21°78′ N, 111°23′ E) in the Guangdong Province, China. The larvae were subsequently reared on fresh *A. sinensis* leaves and kept under laboratory conditions of 25 ± 1 °C, 80 ± 2% relative humidity, and a photoperiod of 14L:10D. Upon reaching maturity, the larvae were transferred to a plastic basin with a lid that had a 5 × 5 cm hole in the middle, and sandy soil (3–4 cm) was spread for pupation. The newly emerged adults were fed with a 10% saccharose solution for feeding.

### 4.2. Scanning Electron Microscopy (SEM)

The antennae, proboscis, labial palps, foreleg, midleg, and hindleg of 4-day-old female and male moths were excised from the base using tweezers under a stereomicroscope (Olympus SZ61, Tokyo, Japan). The surface scales of the tissues were removed using double-sided glue and rinsed with 70% alcohol in a Petri dish. Next, the tissues were subjected to ultrasonic cleaning to remove surface impurities, followed by placement in separate 1.5 mL centrifuge tubes containing 70% alcohol. The samples were then subjected to a series of alcohol dehydration steps (70%, 80%, 95%, and 100%) before being dried using a critical point drier (LEICA EM CPD, Wetzlar, Germany) and treated with a gold spray. The samples were then affixed onto SEM stubs using a double graphite adhesive tape for subsequent observation. Scanning was carried out using a Hitachi SU8010 scanning electron microscope (Hitachi, Tokyo, Japan) at a voltage range of 3–10 kV.

### 4.3. Terminology

The process of identifying and categorizing sensillum types was conducted following the methodology of Schneider [45]. Sensilla were distinguished based on their external morphology in consecutive SEM micrographs. Subsequently, the subtypes of sensilla in each group were classified based on their size, the presence of a basal socket, and their external morphology.

### 4.4. Statistical Analysis

The digital images were adjusted using Adobe Photoshop 2022 (Adobe Systems, San Jose, CA, USA), merely to remove extraneous impurities in the background, and the brightness and contrast were calibrated. All figures were then compiled using Adobe Illustrator 2022 (Adobe Systems). The sizes (length and width, *n* = 20) of the sensilla were measured with the aid of Adobe Photoshop 2022 (Adobe Systems) and subsequently analyzed using Microsoft Excel (Microsoft Corporation, Redmond, WA, USA). All statistical analyses were carried out using IBM Corporation’s SPSS Statistics 26.0 software (Armonk, NY, USA). The length and width of the antennae, sensilla size, and the number of sensilla on the ventral and dorsal sides were subjected to Student’s *t*-test. A significance level of *p* < 0.05 was considered statistically significant.

## 5. Conclusions

In summary, our investigation revealed a total of seven types of sensilla on the antennae, four types on the proboscis, four types on the labial palps, and five types on the legs of *H. vitessoides*. Detailed information on the size, distribution, and abundance of each type of sensilla was also recorded. This study provides valuable morphological data to help further research on *H. vitessoides* and lepidoptera taxonomy. Future investigations should employ advanced techniques such as transmission electron microscopy to obtain more precise descriptions and facilitate molecular biology and electrophysiology-based studies on the function of sensilla in the economically import lepidopteran pest *H. vitessoides.*

## Figures and Tables

**Table 1:** Size of antennal sensilla (length, basal diameter, width of socket, numbers of spines). Data represent means ± SEs.

Sensilla Type	Sex	Length (μm)	Basal Diameter (μm)	Width of Socket (μm)	Numbers of Spines
Sensilla trichodea I	Female	31.09 ± 3.42 *	2.18 ± 0.27	4.14 ± 0.39	
	Male	28.76 ± 2.38	2.3 ± 0.25	3.99 ± 0.42	
Sensilla trichodea II	Female	24.7 ± 3.75 *	1.89 ± 0.38 *	4.17 ± 0.47 *	
	Male	21.13 ± 2.45	1.69 ± 0.23	3.57 ± 0.36	
Sensilla coeloconica I	Female	4.45 ± 0.67		8.6 ± 1.42	8–11
	Male	3.64 ± 0.63		8.23 ± 0.95	8–14
Sensilla coeloconica II	Female	5.06 ± 1.05		5.78 ± 1.19	
	Male	4.14 ± 0.54		7.61 ± 0.89	
Sensilla chaetica	Female	49.21 ± 8.11	3.45 ± 0.51	6.79 ± 0.98	
	Male	57.82 ± 7.5 *	3.22 ± 0.41	7.21 ± 0.78	
Sensilla styloconica	Female	21.54 ± 2.73	6.57 ± 1.22		
	Male	30.04 ± 7.44 *	6.85 ± 1.6		
Sensilla basiconica I	Female	6.03 ± 1.51	1.6 ± 0.25	3.96 ± 0.62	
	Male	4.73 ± 1.52	1.59 ± 0.24	3.42 ± 0.7	
Sensilla basiconica II	Female	19.63 ± 1.96	2.31 ± 0.23 *	3.96 ± 0.35	
	Male	20.74 ± 3.13	2.05 ± 0.41	3.96 ± 0.65	
Capitate sensilla basiconica	Female	4.49 ± 0.48	1.95 ± 0.31	4.13 ± 0.52	
	Male	4.58 ± 0.8	2.05 ± 0.37	4.46 ± 0.68	
Sensilla squamiformia	Female	46.73 ± 3.05	1.65 ± 0.43	3.02 ± 0.57	
	Male	50.24 ± 7.6	2.12 ± 0.45	5.49 ± 1.26	
Böhm bristles	Female	23.46 ± 5.68 *	2.11 ± 0.28 *		
	Male	16.95 ± 3.83	1.7 ± 0.47		

* Significant differences between genders (analyzed by independent *t*-test, *p* < 0.05).

## Data Availability

All data are contained within the article and Supplementary Materials.

## Supplementary Materials

The following supporting information can be downloaded at: https://www.mdpi.com/article/10.3390/insects14080687/s1. Figure S1: Physiological development of *Heortia vitessoides.*
