# Supplementary material for: Ultrastructural and Descriptive Study on the Adult Body Surface of Heortia vitessoides (Lepidoptera: Crambidae)
Source: Insects. 2023 Aug 3;14(8):687. doi: 10.3390/insects14080687 (PMC10455263; doi:10.3390/insects14080687)
Supplement: Supplementary file 1 [file insects-14-00687-s001.zip › insects-2502636-supplementary.pdf]

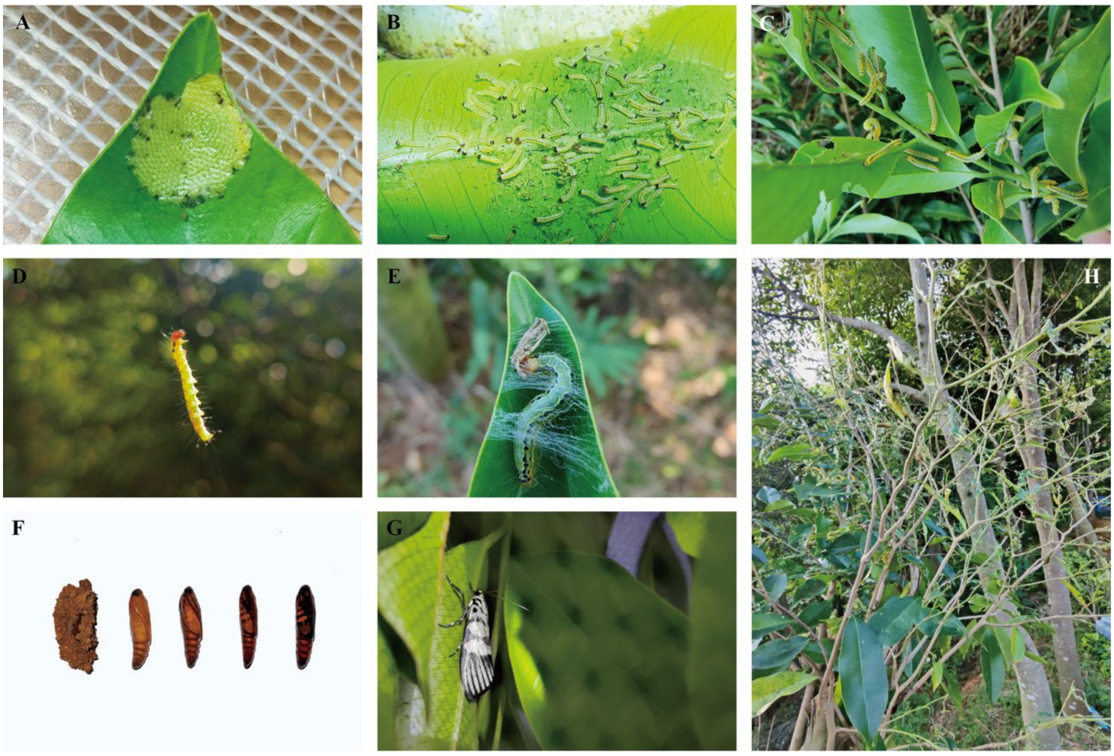

Figure S1. Physiological development of *Heortia vitessoides*. (**A**) Egg masses gathered at the abaxial tip of a juvenile leaf; (**B**) second instar *H. vitessoides* larvae gathering to feed on leaf flesh; (**C**) third instar larvae dispersing to feed on the entire leaf; (**D**) fifth instar larvae descending along a silk thread to undergo pupation in the soil. (**E**) fifth instar larvae cocooning on the leaf to pupate; (**F**) pupae at different stages. The gradual darkening of the pupa's color and the emergence of wing patterns signify the completion of development and its readiness to undergo eclosion. (**G**) *H. vitessoides* adults resting on the leaves of *Aquilaria sinensis*; (**H**) the leaves on the branches of *A. sinensis* are completely consumed by the larvae of *H. vitessoides*.
